# Isolated relapse of multiple myeloma in the central nervous system post autologous hematopoietic stem cell transplantation: a case report and literature review

**DOI:** 10.3389/fonc.2025.1628297

**Published:** 2025-07-08

**Authors:** Ying Zhou, Qian Shen, Jie Zhang, Li Zhu, Qi Jiang, Xiaobing Miao

**Affiliations:** ^1^ Department of Hematology, Affiliated Tumor Hospital of Nantong University, Nantong, Jiangsu, China; ^2^ Department of Pathology, Affiliated Tumor Hospital of Nantong University, Nantong, Jiangsu, China

**Keywords:** multiple myeloma, central nervous system, relapse, treatment, case report

## Abstract

This case report retrospectively evaluated the diagnosis and treatment of a multiple myeloma (MM) patient with early relapse in the central nervous system (CNS) post autologous hematopoietic stem cell transplantation (AHSCT). We also performed a literature review of treatment options for patients with CNS myeloma. The patient was diagnosed with isolated CNS relapse of multiple myeloma one month after AHSCT, without other extramedullary lesions and with normal blood, urine, and bone marrow profiles. The patient responded better to combination therapy involving doxorubicin liposomes and a triple intrathecal injection of dexamethasone plus methotrexate plus cytarabine. However, regimens based on the daratumumab, pomalidomide, ixazomib, and selinexor failed to achieve sustained remission in this patient. Seventeen months after experiencing CNS relapse, the patient died because of disease progression. Currently, there is no standard treatment strategy for CNS myeloma and the overall prognosis is still poor.

## 1 Introduction

Multiple myeloma (MM) is a hematopoietic malignancy characterized by clonal proliferation of plasma cells in the bone marrow. Central nervous system (CNS) involvement in MM is a rare extramedullary manifestation. The incidence of CNS myeloma is less than 1% worldwide (1–3) and 1.2-1.6% in China (4–7). Currently, there is no standard effective treatment regimen for CNS myeloma. Traditional treatment approaches include systemic chemotherapy, local radiotherapy, intrathecal injections, and autologous hematopoietic stem cell transplantation (AHSCT). However, these methods are ineffective and only provide short-term survival benefits. In recent years, novel targeted drugs such as daratumumab and pomalidomide, as well as Chimeric Antigen Receptor T-Cell (CAR-T) immunotherapy have shown better ability to penetrate the blood-brain barrier (BBB) and have been considered for the treatment of CNS myeloma. In this case report, we retrospectively analyzed the diagnosis and therapeutic management of a MM patient with early CNS relapse following AHSCT and conducted a literature review of the treatment options in CNS myeloma.

## 2 Case report

A 52-year-old male with lower back pain was referred to our hospital in May 2022. Positron emission tomography/computed tomographic (PET-CT) scan demonstrated multiple bone destruction in the sternum, ribs, spine, and pelvis, formation of soft tissue masses in some of the affected areas, and unevenly increased fluorodeoxyglucose metabolism. Bone marrow smear showed a high percentage of immature plasma cells (73.5%) indicating a plasma cell disorder. Blood examinations showed anemia, renal insufficiency, and elevated serum lactate dehydrogenase (271 U/L, range: 106~211 U/L). Serum β2-microglobulin was elevated at 21.3 mg/L (range: 0~2 mg/L). The serum immunofixation electrophoresis showed a lambda light chain type. The patient declined cytogenetic examination for financial reasons. The patient was diagnosed with MM, specifically lambda light chain type and R-ISS stage III (high-risk).

The patient was administered two cycles of the PAD regimen (2.6 mg bortezomib on days 1, 4, 8, 11; 40 mg doxorubicin liposome on day 2; 20 mg dexamethasone on days 1-2, 4-5, 8-9, 11-12), starting from May 13, 2022. After normalization of renal function, the patient was administered two additional cycles of the VRd regimen (2.6 mg bortezomib on days 1, 4, 8, 11; 25 mg lenalidomide on days 1-21; 20 mg dexamethasone on days 1-2, 4-5, 8-9, 11-12). Complete remission was achieved after four cycles of induction therapy. The patient underwent high-dose melphalan (200 mg/m^2^) therapy with AHSCT at 6 months after initial diagnosis.

In about a month or more after transplantation (January 2023), the patient experienced progressively worsening lumbar and leg pain accompanied by right upper eyelid ptosis. The PET-CT scan indicated multiple slightly high-density foci in the left frontal lobe, bilateral temporal lobes, sella region, and cisterna magna, as well as patchy foci in the left ethmoid sinus. All these regions of the brain showed varying degrees of increased glucose metabolism. Scattered increases in glucose metabolism were also observed in the spine and nerve roots such as L4 left and S1-3. These were not observed in the previous scans. Reduced glucose metabolism of the right ocular muscles compared to the contralateral side indicated potential damage to the oculomotor nerve. Cerebrospinal fluid (CSF) analysis demonstrated significantly higher total cell counts (103×10^6^/L) and total protein levels (2757 mg/L). The flow cytometry results showed a significantly high percentage of clonal plasma cells (28%) in the CSF characterized by the expression of CD38, CD138, and CD81 with dim CD45 expression and lacking CD19, CD117, and CD27. Re-examination of blood and urine immunoglobulins, serum immune-fixed electrophoresis, and bone marrow demonstrated normal results. The patient did not exhibit any infection-related symptoms. The clinical history, PET-CT, and CSF analysis results strongly indicated the presence of CNS myeloma.

**Figure 1:**
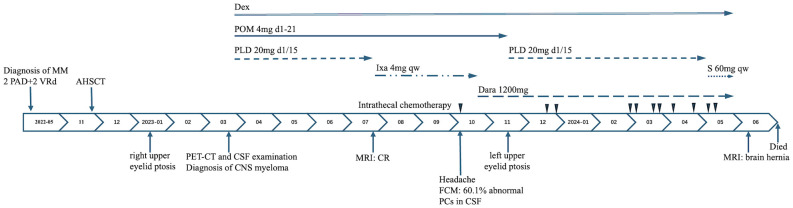
Clinical course from the initial diagnosis of multiple myeloma. MM, multiple myeloma; PAD, bortezomib, doxorubicin liposome, dexamethasone; VRd, bortezomib, lenalidomide, dexamethasone; AHSCT, autologous hematopoietic stem cell transplantation; PET-CT, positron emission tomography/computed tomographic scan; CSF, cerebrospinal fluid; CNS, central nervous system; MRI, magnetic resonance imaging; CR, complete remission; FCM, flow cytometry; PCs, plasma cells; Dex, dexamethasone; POM, pomalidomide; PLD, doxorubicin liposome; Ixa, ixazomib; S, Selinexor; Dara, daratumumab; qw, once a week.

**Figure 2:**
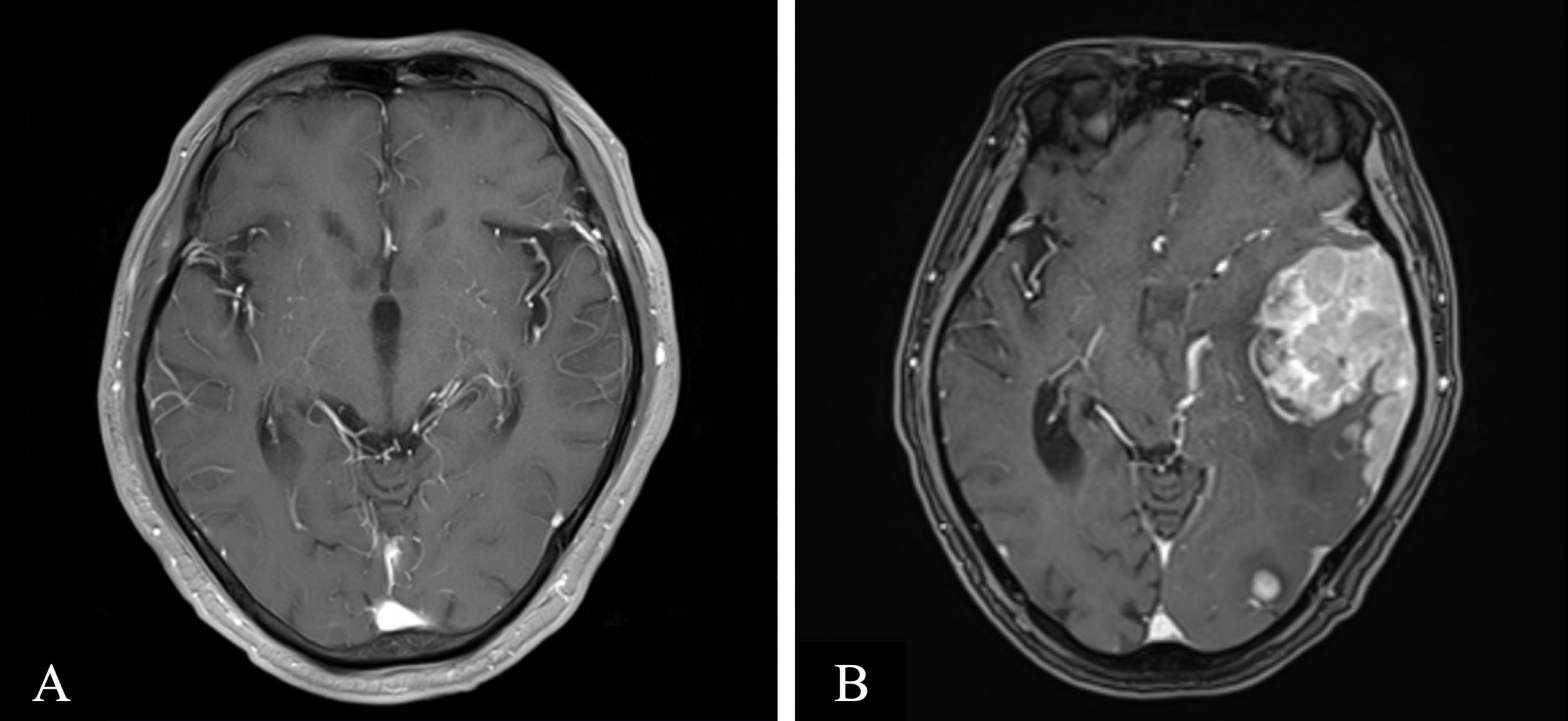
Axial contrasted T1-weighted magnetic resonance (MR) imaging. **(A)** MR imaging on 2023-07–26 revealed few lacunars ischemic foci in the brain parenchyma, otherwise unremarkable **(B)** MR imaging on 2024-06–11 revealed multiple lesions in the left cerebral hemisphere, suggesting tumor infiltration, affecting the corresponding brain parenchyma and adjacent meninges, with perilesional edema in some lesions and a nodule at the lower edge of the fourth ventricle, indicating tumor infiltration and the formation of a brain hernia.

The patient received four cycles of treatment with doxorubicin liposome (20 mg on days 1 and 15), pomalidomide (4 mg daily for 21 days), and dexamethasone (20 mg on days 1–4 and 15-18) from 27^th^ March to 28^th^ June 2023 (**Figure 1**). This significantly alleviated the lumbar and leg pain and right eyelid ptosis of the patient. A plain and contrast-enhanced brain magnetic resonance imaging (MRI) scan conducted on 26^th^ July 2023 was unremarkable but showed a few lacunar ischemic foci in the brain parenchyma (**Figure 2**). Combinatorial pomalidomide regimen was effective in managing the patient’s condition, but the patient declined further inpatient chemotherapy. Therefore, he was transitioned from doxorubicin liposome to oral ixazomib. Between 27^th^ July to 23^rd^ September 2023, the patient underwent three cycles of oral treatment with ixazomib, pomalidomide, and dexamethasone. In October 2023, the patient developed headaches. Flow cytometry results of the lumbar puncture CSF demonstrated a high percentage of abnormal plasma cells (60.1%). Since daratumumab has shown better ability to cross the BBB and is covered by insurance, we substituted ixazomib with daratumumab. On 23^rd^ October 2023, the patient received one cycle of treatment with daratumumab, pomalidomide, and dexamethasone. On 18^th^ November 2023, the patient developed left upper eyelid ptosis. Between 20^th^ November 2023 to 8^th^ April 2024, the patient received five cycles of treatment with daratumumab, doxorubicin liposome, and dexamethasone. During the intervals between chemotherapy cycles, the patient underwent lumbar puncture with intrathecal injections of dexamethasone (5 mg) plus methotrexate (15 mg) plus cytarabine (50 mg). On 10^th^ May 2024, the patient received a course of treatment with daratumumab, selinexor (60 mg once weekly), and dexamethasone. In late May 2024, the patient developed aphasia and right lower limb motor dysfunction. Plain and contrast-enhanced brain MRI scan on 11^th^ June 2024 revealed multiple lesions in the left cerebral hemisphere suggesting tumor infiltration affecting the corresponding brain parenchyma and adjacent meninges, and perilesional edema in some lesions and a nodule at the lower edge of the fourth ventricle indicating tumor infiltration and formation of brain hernia (**Figure 2**). The patient declined further treatment and died within one month after discharge.

## 3 Discussion

The prognosis of patients with recurrent MM in the CNS is extremely poor, with a median overall survival (OS) of 3–6 months (8, 9). Therefore, there is an urgent need for discovering effective treatment strategies that can improve the prognosis of this patient group. Because of the low incidence rates of CNS myeloma, it is difficult to conduct large-scale, high-quality, prospective studies. Therefore, existing research data is obtained from case reports and single-center, small-sample, retrospective analyses (10). Furthermore, BBB is a significant challenge in the treatment of CNS myeloma because it prevents most commonly used anti-myeloma drugs from entering the CNS to exert their effects.

### 3.1 Local therapy

#### 3.1.1 Intrathecal chemotherapy

Application of the classic triplet intrathecal regimen (dexamethasone + methotrexate ± cytarabine) in CNS myeloma is controversial because of limited evidence regarding the sensitivity of myeloma cells to methotrexate or cytarabine. Intrathecal injections are typically used in combination with systemic therapy and their efficacy as monotherapy is yet to be proven. In a multicenter retrospective study in Japan, survival analysis showed no significant difference in OS between the intrathecal injection group (n=31) and the non-intrathecal injection group (n=29), while multivariate analysis suggested a trend towards improved OS with intrathecal treatment (*p*=0.05) (9). Our case showed a progressive reduction in the number of cells and total protein levels in the CSF after receiving the intrathecal injection. Our case study results, and existing research reports suggest that the classic triplet intrathecal regimen rapidly clears myeloma cells from the CSF but should still be used in combination with systemic medication or as a bridge therapy before systemic treatment.

In addition to the classic triple intrathecal injection regimen, thiotepa has also been investigated as an alternative agent in the intrathecal treatment of CNS myeloma. A single-center retrospective study in 2021 evaluated the efficacy and safety of thiotepa intrathecal injection combined with methylprednisolone (40 mg once weekly) in the treatment of 13 patients with CNS myeloma (11). Eleven patients experienced relief from neurological symptoms and 5 patients achieved cytological clearance of plasma cells from the CSF. The median OS for all patients was 17 months. Furthermore, the study confirmed safety of thiotepa as an intrathecal treatment. Therefore, future research directions include investigating the use of intrathecal injection of drugs with anti-myeloma activity, including novel targeted drugs, for the treatment of CNS myeloma.

#### 3.1.2 Radiotherapy

Myeloma cells are highly sensitive to radiation. Therefore, radiotherapy is a cornerstone in the treatment of solitary bone plasmacytomas and solitary extramedullary plasmacytomas. Radiotherapy is also used in the treatment of CNS myeloma. A multicenter retrospective study in Japan demonstrated that there was no significant difference in the OS between the radiotherapy group (n=29) and the non-radiotherapy group (n=31), but multivariate analysis showed that radiotherapy significantly improved the OS of patients with CNS myeloma (*p*=0.0009), and landmark analysis further confirmed this conclusion (9). The toxic side effects of whole-brain radiotherapy limit its clinical application, but there is increasing evidence that modern radiotherapy techniques achieve optimal responses without significant toxicity in CNS myeloma (12). Therefore, application of radiotherapy in CNS myeloma warrants further research.

### 3.2 Systemic therapies

A large multicenter retrospective study by Jurczyszyn et al. reported that patients with CNS myeloma receiving systemic treatment showed better prognosis with a median OS of 12 months compared to a median OS of only 3 months for patients receiving other treatments such as surgery + radiotherapy, radiotherapy + intrathecal injection, hormone monotherapy, radiotherapy, and intrathecal injection; except for systemic treatment, survival rates of patients receiving other treatments were comparable with untreated patients (8). A nationwide multicenter retrospective study in Japan by Yamashita et al. also reported that patients receiving combination therapy (lenalidomide + radiotherapy + intrathecal injection) showed better prognosis than those receiving only one or two of these treatments (*p*=0.002) (9). These data suggest that local treatment methods such as intrathecal injection and radiotherapy are not effective in suppressing the development of CNS myeloma, and systemic treatment is required for improving the prognosis of patients with CNS myeloma.

#### 3.2.1 Immunomodulators

Immunomodulatory drugs have shown better ability to penetrate the BBB. After oral administration, thalidomide, lenalidomide, and pomalidomide showed CSF concentrations of 42%, 11%, and 39%, respectively (13). Furthermore, several case reports have reported improved efficacy and outcomes in patients with CNS myeloma treated with thalidomide (14), lenalidomide (9), and pomalidomide (15, 16).

#### 3.2.2 Proteasome inhibitors

Proteasome inhibitors such as bortezomib, carfilzomib, and ixazomib cannot penetrate the BBB (17). However, bortezomib enhances the sensitivity of CNS myeloma cells to radiotherapy and chemotherapy, probably because of pathological changes such as inflammation increasing the permeability of the BBB (10). Currently, there are no published reports of CNS myeloma patients being treated with carfilzomib and ixazomib. Marizomib, a newer reversible proteasome inhibitor, has shown potential efficacy in the treatment of CNS myeloma and improved penetration of BBB with a CSF concentration of 30% (13, 18).

#### 3.2.3 CD38 monoclonal antibodies

Vercruyssen et al. analyzed plasma and CSF samples from a patient with NK/T-cell lymphoma and CNS involvement undergoing daratumumab monotherapy and confirmed that daratumumab penetrated the BBB (19). However, Zajec et al. reported that although daratumumab penetrated the BBB, its concentration in the CSF was insufficient to produce a significant therapeutic effect (20). Recently, a couple of case reports have demonstrated that combination of daratumumab with intrathecal chemotherapy is effective in treating CNS myeloma (21, 22).

#### 3.2.4 Other drugs

Selinexor has been shown to cross the BBB in animal models with a CSF to plasma concentration ratio of 0.72 in rats (23). In 16 patients with extramedullary myeloma treated with Selinexor, the overall response rate was 56% (24), thereby suggesting its potential application in the treatment of CNS myeloma. As a selective BCL-2 inhibitor, venetoclax can induce apoptosis in myeloma cells, with those harboring t (11, 18) translocation exhibiting particular sensitivity to venetoclax. Venetoclax can also cross the BBB (25), but there are no reported cases of its use in treating CNS myeloma. Currently, there is no data regarding the CNS penetration and treatment efficacies of both isatuximab and elotuzumab in CNS myeloma. Mutlu et al. reported a male MM patient with CNS myeloma who was successfully treated with Elranatamab, a humanized bispecific antibody targeting B-cell maturation antigen (BCMA) and CD3-expressing T cells (26).

#### 3.2.5 AHSCT

AHSCT is a first-line treatment option for newly diagnosed MM. High-dose melphalan is the classic conditioning regimen for AHSCT but demonstrates only 10% penetration into the CNS (27). Therefore, high-dose melphalan is not the preferred conditioning regimen for CNS myeloma. The CNS penetration rates for busulfan, carmustine, cyclophosphamide, and etoposide are >80%, 15-70%, 20-30%, and <5%, respectively (27). The BBB penetration rate for thiotepa is 100% (28). In newly diagnosed MM, the conditioning regimen of thiotepa and busulfan demonstrated longer progression-free survival (41.5 months *vs*. 24.4 months) and reduced risk of extramedullary lesions (HR = 0.43) compared to the high-dose melphalan (29). For patients with CNS myeloma undergoing AHSCT, conditioning regimens should include drugs with high CNS penetration rates. Further studies are necessary to determine whether AHSCT improves the prognosis of patients with CNS myeloma.

#### 3.2.6 CAR T-cell therapy

Despite limited number of reported cases, CAR T-cell therapy has shown promising results for CNS myeloma (16, 30–32). CAR T-cells can cross the BBB. Wang et al. evaluated the results of BCMA CAR T-cell therapy in 4 patients with CNS myeloma and reported that 3 achieved complete remission (32). The main side effect of BCMA CAR T-cell therapy was grade 1–2 cytokine release syndrome, but neurotoxicity was not observed. This suggested that BCMA CAR-T therapy was a safe and feasible option for CNS myeloma. Based on a case report, Wang et al. recently reported that BCMA CAR T-cell therapy was safe and effective for patients with CNS myeloma who have multiple extramedullary lesions (31). CAR T-cell therapy is expected to be an effective treatment option for patients with CNS myeloma.

Our patient experienced a relapse in the CNS. Combined treatment with doxorubicin liposome and pomalidomide was effective initially. Subsequently, the treatment was switched to ixazomib, daratumumab, and selinexor in sequence, but disease progression was observed. Switching to combined treatment with doxorubicin liposome and intrathecal therapy again alleviated the clinical symptoms. This suggests that pomalidomide, ixazomib, daratumumab, and selinexor were not able to achieve durable remission in this patient. However, the patient survived for 17 months after the diagnosis of CNS myeloma. This was better than the median survival of 6 months reported in literature. In the era of new drugs, a critical challenge in the treatment of CNS myeloma patients is the selection of potentially effective individualized regimens to further improve their prognosis. If clinical conditions allow, aggressive approaches such as bispecific antibodies, BCMA CAR-T, and transplantation should be considered for treating patients with CNS myeloma.

## 4 Conclusions

In summary, CNS myeloma is associated with low incidence rates and poor prognosis. Currently, there is no standard and effective treatment regimen. However, combining systemic treatments that can penetrate the BBB with local treatment methods such as intrathecal injection and radiotherapy is critical for improving the prognosis of patients with CNS myeloma.

## Data Availability

The original contributions presented in the study are included in the article/supplementary material. Further inquiries can be directed to the corresponding author.
